# The HO-1/bilirubin axis in cardiovascular protection: critical evidence for modulation by plant metabolites and traditional multi-component formulations used in Chinese medicine

**DOI:** 10.3389/fphar.2026.1902935

**Published:** 2026-09-21

**Authors:** Rui Zhang, Jia Wang, Mengyuan Liu, Shan Wu, Wenxiu Qin, Jianghan Qi, Guo Yu, Ye Hou, Jingqing Hu

**Affiliations:** 1 Tianjin University of Traditional Chinese Medicine, Tianjin, China; 2 The Second Affiliated Hospital of Tianjin University of Traditional Chinese Medicine, Tianjin, China; 3 Nanjing University of Chinese Medicine, Nanjing, China; 4 Hubei University of Chinese Medicine, Wuhan, China; 5 Liaoning University of Traditional Chinese Medicine, Shenyang, China

**Keywords:** bilirubin, cardiovascular disease, Chinese medicine, heme oxygenase-1, plant metabolites

## Abstract

Bilirubin was long regarded primarily as a waste product of haem catabolism, but accumulating experimental and epidemiological evidence suggests that, within an appropriate concentration range, it may contribute to cardiovascular protection through antioxidant, anti-inflammatory, endothelial, metabolic, and mitochondrial effects. Haem oxygenase-1 (HO-1) is a key inducible enzyme in bilirubin formation because it converts haem to biliverdin, which is subsequently reduced to bilirubin. However, HO-1 activation also produces carbon monoxide and ferrous iron, and increased HO-1 expression alone does not demonstrate enhanced bilirubin production or bilirubin-mediated protection. This critical narrative review first examines the biological basis and evidential limits of bilirubin-related cardiovascular protection and then evaluates whether selected plant metabolites and traditional multi-component formulations used in Chinese medicine engage the HO-1/bilirubin axis in cardiovascular models. Curcumin provides the most direct evidence of pathway engagement because HO activity, circulating bilirubin, pharmacological inhibition, and HO-1 silencing were assessed. Dihydromyricetin shows HO-1-dependent vascular protection, although bilirubin was not measured. For most other metabolites and formulations, the evidence is limited to Nrf2/HO-1-associated signalling in preclinical models. Material characterisation, dose justification, bioavailability, pharmacokinetics, safety assessment, and direct measurement of haem-catabolic products remain inconsistent. The available data support the HO-1/bilirubin axis as a biologically plausible link between selected interventions used in Chinese medicine and cardiovascular protection, but direct bilirubin-specific causality remains unproven. Future studies should combine standardised materials, exposure-confirmed dosing, direct quantification of bilirubin and biliverdin, *HMOX1*/BVRA manipulation, and product-specific rescue experiments.

## 1 Introduction

Bilirubin was long regarded mainly as an end product of haem catabolism and as a clinical marker of hepatic or haemolytic disease. Accumulating experimental and epidemiological evidence now suggests that, within an appropriate concentration range, bilirubin may also contribute to cardiovascular homeostasis. This change in perspective has renewed interest in the enzymatic pathway responsible for bilirubin formation. Haem oxygenase-1 (HO-1) is particularly relevant because it removes pro-oxidant haem and generates biliverdin, carbon monoxide, and ferrous iron; biliverdin is subsequently reduced to bilirubin by biliverdin reductase A. The consequences of HO-1 induction therefore depend not only on bilirubin formation but also on haem removal, biliverdin redox activity, carbon monoxide signalling, and the sequestration or export of released iron (Ryter et al., 2006; Vítek and Tiribelli, 2021). This broader haem-catabolic context is essential when the HO-1/bilirubin axis is considered as a potential cardiovascular pharmacological target.

Botanical drugs and traditional multi-component formulations have long been used in Chinese medicine for symptoms and disorders that include chest discomfort, palpitations, and impaired circulation. Modern pharmacological studies have examined whether these preparations, and metabolites isolated from their constituent materials, influence oxidative, inflammatory, vascular, and myocardial pathways (Hao et al., 2017). Traditional use, however, is not equivalent to experimental evidence. Curcumin, quercetin, berberine, and several other metabolites considered here are widely distributed natural products rather than compounds unique to Chinese medicine. Moreover, traditional multi-component formulations differ in composition, processing, preparation, dose, and quality control. Mechanistic claims must therefore be linked to the specific material and experimental conditions studied.

A central interpretive problem is the frequent assumption that increased *HMOX1* transcription or HO-1 protein expression necessarily indicates greater bilirubin production. In many studies, HO activity and the concentrations of bilirubin, biliverdin, carbon monoxide, and iron-related products were not measured. Even when circulating bilirubin rises, the change may reflect altered hepatic uptake, glucuronidation, biliary excretion, haemolysis, or intestinal metabolism rather than increased HO-1-dependent synthesis. Only a few cardiovascular studies have combined pathway manipulation with direct measurement of haem-catabolic products or rescue experiments capable of supporting product-specific causality (Liu et al., 2015b; Xiao et al., 2018). Interpretation is further limited by non-cardiovascular models, poorly justified concentrations, incomplete material characterisation, and sparse pharmacokinetic and safety data. These issues are especially important in ethnopharmacological research, in which reproducibility depends on accurate material identification and adequate chemical definition (Heinrich et al., 2022). These processes are summarized in Figure 1.

**FIGURE 1:**
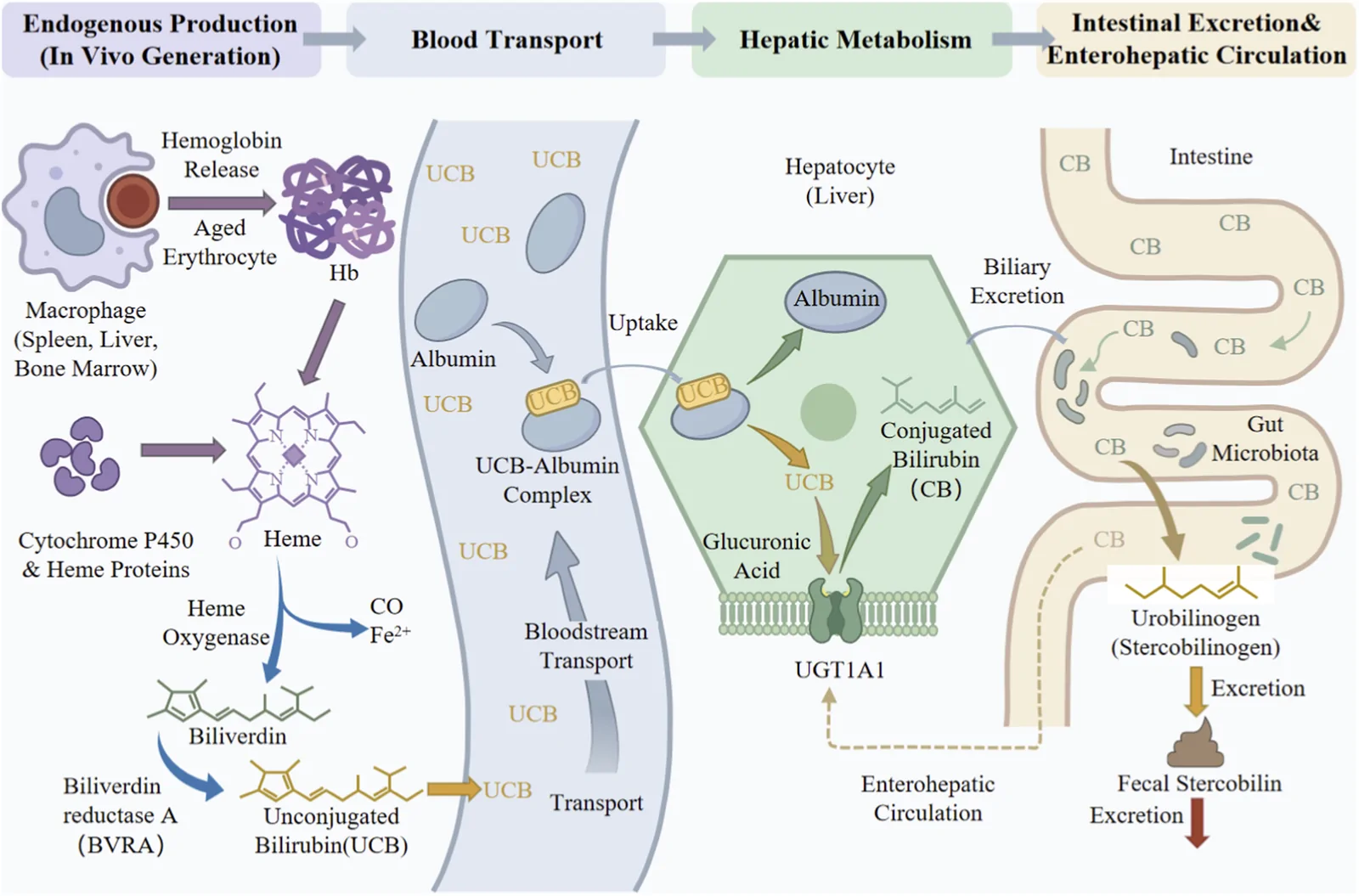
Endogenous production, transport, metabolism, excretion, and enterohepatic circulation of bilirubin. Circulating bilirubin levels are determined not only by haem catabolism but also by hepatic uptake, conjugation, biliary excretion, and intestinal metabolism. Abbreviations: BVRA, biliverdin reductase A; CB, conjugated bilirubin; CO, carbon monoxide; Fe^2+^, ferrous iron; Hb, haemoglobin; UCB, unconjugated bilirubin; UGT1A1, uridine diphosphate glucuronosyltransferase 1A1.

This review addresses the HO-1/bilirubin relationship from two linked perspectives. It first examines the proposed cardiovascular actions of bilirubin and the limits of the mechanistic and clinical evidence. It then evaluates cardiovascular studies of selected plant metabolites and traditional multi-component formulations used in Chinese medicine, focusing on model relevance, material characterisation, dose and duration, pathway-specific controls, HO activity, direct measurement of haem-catabolic products, and causal intervention. The aim is not to infer a common mechanism for Chinese medicine as a whole, but to identify which individual interventions show credible pathway engagement, where the evidence remains associative, and which experiments are still required to establish bilirubin-mediated protection.

### 1.1 Literature search strategy and qualitative evidence appraisal

This article was developed as a structured critical narrative review rather than a systematic review. The initial topic-oriented search was conducted from October 2025 to May 2026 in PubMed, Web of Science Core Collection, Embase, the China National Knowledge Infrastructure (CNKI), and Wanfang Data. Search terms were combined according to the topic under consideration and included “bilirubin,” “biliverdin,” “biliverdin reductase,” “*BVRA*,” “heme oxygenase-1,” “HO-1,” “*HMOX1*,” “cardiovascular disease,” “myocardial injury,” “vascular dysfunction,” “traditional Chinese medicine,” “Chinese herbal medicine,” and the names of the plant metabolites and traditional multi-component formulations discussed in this review. Reference lists of relevant original studies and reviews were also screened.

During revision, documented PubMed searches were run in July 2026 to identify recent cardiovascular, pharmacological, and mechanistic evidence on the HO-1/haem-catabolic pathway. Broad searches mapped the literature on HO-1, bilirubin, carbon monoxide, iron homeostasis, and cardiovascular disease; these searches were not treated as a formal systematic-review screening cohort. Targeted update searches were also conducted for metabolites whose original supporting evidence was indirect or derived from non-cardiovascular models. Search dates, purposes, complete PubMed strings, and retrieved-record counts are reported in Supplementary Table S1.

Original studies were prioritised when they examined: (1) the HO-1/haem-catabolic pathway in a cardiovascular model; (2) a plant metabolite, botanical preparation, or traditional multi-component formulation in a cardiovascular disease model; or (3) pharmacokinetic, bioavailability, safety, or translational questions directly relevant to the reviewed interventions. Non-cardiovascular studies were not treated as direct cardiovascular evidence and were retained only when they supplied otherwise unavailable mechanistic or safety information. Reviews were used mainly for biochemical and pharmacological background. Duplicate publications, commentaries, and studies with insufficient relevance to the revised scope were excluded from the core pharmacological synthesis.

Core pharmacological studies were appraised qualitatively according to model relevance, definition and characterisation of the intervention, dose or concentration, route and duration, adequacy of controls, and measurement of HO-1 expression or activity. Particular attention was paid to direct measurements of bilirubin, biliverdin, carbon monoxide, and iron-related variables, and to the use of pharmacological inhibition, gene silencing, knockout models, or rescue experiments. Evidence was classified as follows: A, direct engagement of the HO-1/bilirubin pathway, defined by either HO-1-specific intervention with direct bilirubin measurement or assessment of HO activity through bilirubin formation; B, direct pharmacological or genetic evidence of HO/HO-1 dependence without bilirubin measurement; C, functional dependence on an upstream regulator, with HO-1 measured as a downstream readout; and D, associative HO-1 evidence without pathway-specific intervention. Non-cardiovascular mechanistic studies were used only as supporting context and were not assigned a core cardiovascular evidence category. Increased HO-1 expression alone was not considered sufficient evidence of a bilirubin-mediated mechanism.

## 2 Overview of the bilirubin and HO-1/bilirubin axis

### 2.1 Production, transport, and elimination of bilirubin

Bilirubin homeostasis is determined by the coordinated processes of production, plasma transport, hepatic conjugation, and intestinal elimination. During the turnover of haemoglobin and other haem-containing proteins, haem oxygenase catalyses the oxidative cleavage of haem to generate biliverdin IXα, carbon monoxide (CO), and iron. Biliverdin IXα is subsequently reduced to bilirubin IXα by biliverdin reductase A (BVRA). This enzymatic sequence constitutes the principal endogenous route of bilirubin formation and provides the biochemical link between haem degradation and the generation of bile pigments (Tenhunen et al., 1968; Adin, 2021; Stec et al., 2022).

Because unconjugated bilirubin is poorly soluble in water, it circulates mainly as a non-covalent complex with serum albumin. At the hepatic sinusoidal surface, bilirubin dissociates from albumin and is taken up by hepatocytes, where uridine diphosphate glucuronosyltransferase 1A1 (UGT1A1) forms water-soluble bilirubin glucuronides. These conjugates are secreted into bile and delivered to the intestine. Gut microbial bilirubin reductase (BilR) reduces bilirubin to urobilinogen, which is converted to stercobilin and related urobilinoids. Most products are eliminated in the faeces, whereas a smaller fraction is reabsorbed through enterohepatic circulation (Petersen et al., 2000; Zhou et al., 2010; Hall et al., 2024).

These downstream processes are particularly relevant when interpreting pharmacological studies of botanical interventions. Circulating bilirubin concentrations reflect the balance between haem degradation, hepatic uptake, glucuronidation, biliary excretion, and intestinal metabolism, rather than HO-1 expression alone. For example, certain plant-derived polyphenolic acids have been shown to inhibit UGT1A1-mediated bilirubin glucuronidation, indicating that an increase in circulating bilirubin may reflect impaired clearance rather than enhanced HO-1-dependent production. Therefore, studies proposing modulation of the HO-1/bilirubin pathway should distinguish increased bilirubin synthesis from altered transport or elimination and should report direct bilirubin measurements whenever possible (Ma et al., 2017; Vítek and Tiribelli, 2021).

### 2.2 Biochemical basis and evidential boundaries of the HO-1/bilirubin axis

Haem oxygenase-1 (HO-1) is the stress-responsive isoform of haem oxygenase and catalyses the rate-limiting oxidative cleavage of haem. Its enzymatic activity simultaneously removes potentially pro-oxidant free haem and generates equimolar biliverdin IXα, CO, and Fe^2+^. BVRA subsequently converts biliverdin IXα to bilirubin IXα. Accordingly, HO-1 induction initiates a broader haem-catabolic response rather than an exclusively bilirubin-producing pathway, and its biological consequences may arise from the combined effects of haem removal, biliverdin/bilirubin formation, CO-dependent signalling, and adaptive iron handling (Tenhunen et al., 1968; Ryter et al., 2006; Gáll et al., 2019; O'Rourke et al., 2024).

Carbon monoxide (CO), one of the non-pigment products of haem catabolism, acts as a concentration-dependent signalling molecule rather than solely as a toxic by-product. At low, controlled concentrations, CO can influence vascular tone through soluble guanylate cyclase/cyclic guanosine monophosphate signalling and potassium channels, although responses vary by vascular bed and experimental setting. In preclinical cardiac models, a CO-releasing molecule or brief CO exposure induced a delayed preconditioning-like response, reduced infarct size, and promoted an anti-apoptotic phenotype (Villamor et al., 2000; Stein et al., 2005; van der Sterren et al., 2011). Because these experiments used exogenous CO or CO-releasing molecules, they establish biological plausibility but do not show that a botanical intervention acts through endogenous HO-1-derived CO unless CO production or CO-dependent signalling is directly measured or manipulated.

The Fe^2+^ released during haem degradation represents another important determinant of the biological outcome of HO-1 induction. Because poorly buffered ferrous iron can promote oxidative reactions, effective cytoprotection requires coordination between haem degradation, iron sequestration, and iron export. Ferritin heavy chain has both ferroxidase and iron-storage functions, and experimental depletion of ferritin heavy chain weakened HO-1-associated resistance to oxidative injury, indicating that ferritin is a functional component of the protective response rather than merely a downstream marker (Cheng et al., 2015). In the heart, genetic disruption of cardiomyocyte ferroportin or of the local hepcidin–ferroportin regulatory axis caused marked disturbances of intracellular iron homeostasis and impaired cardiac metabolic and contractile function (Lakhal-Littleton et al., 2015; Lakhal-Littleton et al., 2016). These findings indicate that the consequences of HO-1 activation depend partly on whether the released iron is appropriately sequestered by ferritin or exported through ferroportin. Accordingly, studies of botanical interventions that report HO-1 induction without assessing ferritin, ferroportin, hepcidin, labile iron, or ferroptosis-related endpoints cannot determine whether altered iron handling contributes to the observed cardiovascular effects.

Bilirubin can scavenge selected reactive species, and the BVRA-mediated conversion of biliverdin back to bilirubin has been proposed to form an antioxidant amplification cycle. However, the quantitative importance and general applicability of this bilirubin–biliverdin redox cycle remain debated. Although experimental depletion of BVRA or bilirubin has supported a cytoprotective role in some cellular systems, subsequent biochemical studies have suggested that bilirubin oxidation does not invariably generate sufficient biliverdin to sustain a universal antioxidant amplification mechanism. The redox cycle should therefore be presented as a proposed and context-dependent mechanism rather than as an established explanation for all HO-1-mediated cytoprotection (Stocker, 2004; Maghzal et al., 2009).

This distinction is essential for evaluating the pharmacological literature reviewed in this article. Increased *HMOX1* transcription or HO-1 protein expression indicates activation of an HO-1-associated stress response, but does not by itself demonstrate increased enzymatic activity, enhanced bilirubin production, or bilirubin-dependent cardioprotection. Evidence for direct involvement of the bilirubin pathway is stronger when studies measure HO activity and biliverdin or bilirubin concentrations, or when pharmacological inhibition, gene silencing, knockout models, or metabolite-rescue experiments demonstrate causal dependence. Accordingly, studies reporting only Nrf2 and/or HO-1 expression are described in this review as showing “HO-1-associated signalling,” whereas the term “bilirubin-mediated” is reserved for studies providing direct metabolic or causal evidence (Ryter et al., 2006; Gáll et al., 2019; Vítek and Tiribelli, 2021).

## 3 Cardiovascular effects of bilirubin: molecular mechanisms and evidence limitations

Although bilirubin has been associated with several potentially cardioprotective actions, the strength of the available evidence varies substantially across experimental settings. Most mechanistic findings have been derived from biochemical, cellular, and animal studies, whereas human evidence is predominantly observational. Moreover, HO-1 induction cannot itself be interpreted as evidence of bilirubin-mediated protection unless bilirubin or biliverdin is directly quantified or the proposed pathway is supported by an appropriate causal intervention. The following sections therefore distinguish experimentally demonstrated actions of bilirubin from broader HO-1-associated mechanisms and epidemiological associations.

### 3.1 Antioxidant effects

Bilirubin can react with selected reactive oxygen and nitrogen species and can interrupt lipid peroxidation, properties that may contribute to cardiovascular protection (Stocker, 2004; Soto Conti, 2021; Zhang et al., 2025). Stocker and colleagues first established bilirubin as a chain-breaking antioxidant, challenging the view that it was only a waste product of haem metabolism. Its conjugated tetrapyrrole structure and redox-active hydrogen atoms permit reactions with singlet oxygen, peroxyl radicals, and peroxynitrite. The efficiency and biological importance of these reactions depend on bilirubin concentration, chemical form, binding state, and the surrounding redox environment (Condezo-Hoyos et al., 2009; Sedlak et al., 2009; Rahimi et al., 2022; Cruz et al., 2023).

The bilirubin–biliverdin redox cycle has been proposed as a means of amplifying antioxidant activity at low bilirubin concentrations. Oxidation of bilirubin can generate biliverdin, which biliverdin reductase (BVR) converts back to bilirubin using NADPH. Although this recycling may operate in some cellular settings, its quantitative contribution is uncertain and should not be assumed to explain all HO-1-mediated cytoprotection. Biliverdin also has concentration-dependent antioxidant activity (Baranano et al., 2002; Maghzal et al., 2009).

The lipophilicity of unconjugated bilirubin favours its partitioning into lipid-rich compartments. In membranes and lipoproteins, bilirubin can interrupt lipid-peroxidation chain reactions and can act with α-tocopherol to limit oxidative damage (Neuzil and Stocker, 1994). Access to mitochondrial membranes may provide similar protection against local lipid peroxidation (Lee et al., 2025). These lipid-phase effects complement, rather than replace, aqueous antioxidant systems such as glutathione (Nocentini et al., 2022).

Bilirubin may also influence cellular antioxidant responses, including Nrf2-related signalling, in selected experimental systems (Vítek et al., 2023; Lee et al., 2025; Zhang et al., 2025). The direction and physiological importance of this effect remain model-dependent. In particular, a rise in HO-1 expression after bilirubin exposure does not establish a self-amplifying HO-1/bilirubin loop, because expression alone does not demonstrate increased enzymatic flux, bilirubin production, or tissue exposure (Abraham and Kappas, 2008; Yao et al., 2024; Lee et al., 2025).

Bilirubin has also been reported to limit NADPH oxidase-dependent ROS production in vascular and immune cells (Idelman et al., 2015; Nocentini et al., 2022; Yuan et al., 2023). These effects are concentration-dependent. Bilirubin values near the upper physiological range or within mild hyperbilirubinaemia have been associated with more favourable cardiometabolic profiles, whereas lower values have been associated with greater metabolic risk (Kudo et al., 2021; El-Eshmawy et al., 2022; Xia et al., 2025). Such associations do not show that experimentally increasing bilirubin would improve cardiovascular outcomes.

Excessive bilirubin exposure is toxic. Very high concentrations are well recognised as a cause of neonatal neurotoxicity, and supraphysiological concentrations can also damage cardiomyocytes *in vitro* by disrupting redox balance and membrane-enzyme function. Any strategy intended to raise endogenous bilirubin must therefore distinguish the mild concentrations associated with potential benefit from concentrations that produce cellular toxicity, while recognising that neonatal and adult cardiovascular exposures are not directly comparable (Kunutsor, 2015; Oz et al., 2015; Shen et al., 2019; Kulkarni et al., 2025).

Taken together, biochemical and preclinical studies support antioxidant actions of bilirubin through radical scavenging, lipid-phase protection, redox recycling in selected contexts, and modulation of cellular antioxidant responses. Differences in concentration, chemical form, model, and tissue exposure nevertheless limit direct extrapolation to cardiovascular therapy.

### 3.2 Anti-inflammatory and immunomodulatory effects

Bilirubin can modulate inflammatory signalling, but its effects depend on concentration, cell type, and experimental context (Novák et al., 2020; Tran et al., 2020; Žiberna et al., 2021; Tani et al., 2026). At concentrations near the physiological range, several studies have reported reduced IκB-α phosphorylation and NF-κB activation, with lower production of inflammatory mediators and reduced NLRP3 inflammasome activity in macrophage-related models (Li et al., 2020; Yao et al., 2020). By contrast, bilirubin activated the NLRP3 inflammasome in microglial models, illustrating that an anti-inflammatory effect cannot be assumed across tissues or exposure ranges (Ercan et al., 2023).

In immune and transplantation models, bilirubin has influenced the survival of activated effector T cells through CD95^−^, p38-MAPK-, Bax-, and caspase-related pathways. These observations may help explain how bilirubin limits excessive T-cell responses, but they have not been consistently reproduced in cardiovascular disease models (Liu et al., 2008; Lee et al., 2016; Kim et al., 2021).

Bilirubin may also affect innate immune recruitment. In vascular endothelial cells, it can reduce ROS generated downstream of VCAM-1 and ICAM-1 signalling, thereby limiting monocyte adhesion and preserving junctional integrity (Vogel et al., 2017).

Macrophage studies have reported shifts towards marker profiles associated with an M2-like state, including higher CD163 and CD206 and lower CD86 expression. These findings should be interpreted cautiously because macrophage states are dynamic and cannot be defined by a small marker set (Jangi et al., 2013; Zhao et al., 2021).

Other non-cardiovascular studies suggest that bilirubin can reduce MHC-II expression and IL-12/IL-23 production, restrain Th17 differentiation and glycolysis, and alter regulatory T-cell or myeloid-derived suppressor-cell responses (Longhi et al., 2017; Kim et al., 2021). These mechanisms are relevant to immune regulation but remain indirect evidence for cardiovascular benefit.

Complement modulation has also been observed outside the cardiovascular system. In an isolated rat-hepatocyte antibody/complement model, unconjugated bilirubin reduced C3 deposition and interfered with C1q binding to IgG, thereby attenuating complement-mediated injury (Basiglio et al., 2007).

Thus, bilirubin can influence NF-κB and inflammasome signalling, immune-cell recruitment, macrophage state, T-cell responses, and complement. Much of this evidence comes from transplantation, autoimmune, neurological, or other non-cardiovascular models. It supports mechanistic plausibility, but not cardiovascular efficacy or clinical benefit on its own.

### 3.3 Vascular protection and metabolic regulation

Bilirubin has been proposed as an endogenous modulator of endothelial function and vascular homeostasis. Higher physiological concentrations have been associated with a lower burden of vascular disease, but these associations are not proof of causality (Calay and Mason, 2014; Maruhashi et al., 2019). Experimental work nevertheless identifies several plausible vascular actions.

By scavenging superoxide, bilirubin may preserve nitric oxide (NO) bioavailability. Experimental studies have also linked the haem oxygenase/bilirubin system to PPARα/AMPK signalling and endothelial nitric oxide synthase phosphorylation, changes that may favour vasodilation and restrain platelet and vascular smooth-muscle-cell activation (Ndisang et al., 2009; Drummond et al., 2019). Bilirubin may also reduce endothelin-1 production by limiting oxidative and NF-κB-related signalling (Vogel et al., 2017).

Bilirubin-related antioxidant and anti-inflammatory effects may help preserve endothelial barrier function and limit vascular remodelling. Evidence includes reduced adhesion-molecule signalling and monocyte recruitment in vascular models, together with broader observations of lower endothelial injury and smooth-muscle-cell activation (Calay and Mason, 2014; Vogel et al., 2017). However, the models, bilirubin formulations, and exposure ranges differ substantially, so these findings should not be treated as a single established vascular mechanism.

Experimental evidence also suggests that bilirubin can act as a metabolic signal through peroxisome proliferator-activated receptor alpha (PPARα) (Bulmer et al., 2013; Stec et al., 2016). Binding and transcriptional studies indicate interaction with the PPARα ligand-binding domain, with a reported dissociation constant of approximately 5.13 μM, and recruitment of transcriptional co-activators (Hong et al., 2019; Gordon et al., 2021).

PPARα-related responses to bilirubin include increased expression of genes involved in fatty-acid oxidation, such as CPT1 and ACOX1, and reduced expression of lipogenic targets such as SCD1. Bilirubin has also been associated with higher lipoprotein lipase expression and lower hepatic very-low-density lipoprotein production (Liu et al., 2015a; Hong et al., 2019). The relative contribution of direct PPARα activation, rather than secondary changes in haem metabolism, remains uncertain.

The identification of BilR has clarified a major step in intestinal bilirubin metabolism. BilR converts bilirubin to urobilinogen, which is subsequently oxidised to urobilin or stercobilin, while a fraction undergoes reabsorption and enterohepatic circulation (Hall et al., 2024; Dufault-Thompson et al., 2024). Altered microbial bilirubin reduction has been linked to obesity and metabolic syndrome, but the direction of causality and the clinical relevance of BilR activity remain unresolved (Vítek and Tiribelli, 2025).

These observations have prompted interest in the liver–gut bilirubin axis as a metabolic target, but therapeutic manipulation of microbial bilirubin reduction remains unvalidated. Bilirubin may also influence bile-acid synthesis, biliary excretion, and intestinal lipid handling through nuclear receptors including CAR and PXR (Vítek and Tiribelli, 2025). In experimental obesity, bilirubin has additionally been associated with white-adipose-tissue browning, increased adiponectin, and lower pro-inflammatory adipokine production (Takei et al., 2019).

The available data therefore support possible effects on endothelial signalling, vascular remodelling, and lipid metabolism. Human evidence is still largely observational, and residual confounding, reverse causation, bilirubin fraction, and the absence of controlled bilirubin-modifying interventions limit causal interpretation.

### 3.4 Myocardial protection

Preclinical studies suggest that bilirubin may limit myocardial ischaemia–reperfusion injury by preserving mitochondrial membranes and function (Paradies et al., 2018; Kim et al., 2024). Unconjugated bilirubin can partition into cardiolipin-rich membrane domains, where reduced lipid peroxidation may restrain opening of the mitochondrial permeability transition pore, cytochrome-c release, and apoptosis (Lee et al., 2025). These mechanisms remain dependent on bilirubin form, concentration, and tissue exposure.

PINK1/Parkin-associated mitophagy has been proposed as another possible mechanism. Bilirubin nanoparticles promoted PINK1-mediated mitophagy in a dry-eye model (Huang et al., 2024), but this is non-cardiovascular evidence and does not establish the same pathway in myocardium. Direct cardiac experiments are needed before mitophagy can be regarded as a bilirubin-mediated cardioprotective mechanism.

Mitochondrial protection may therefore complement the antioxidant actions discussed above, but the evidence currently supports a broader preclinical response to myocardial injury rather than a clinically established bilirubin mechanism.

Observational studies have reported inverse associations between serum bilirubin and atherosclerosis, coronary heart disease, or ischaemic stroke, and some associations persist after adjustment for conventional risk factors (Kunutsor, 2015; Thakkar et al., 2019; Su et al., 2023; Mahadevan et al., 2024). These studies do not show that raising bilirubin prevents cardiovascular events. Hepatic function, haemolysis, medication use, nutrition, systemic inflammation, and differences between total and direct bilirubin may all contribute to the observed relationships.

In the PragueOHCA cohort, patients resuscitated after out-of-hospital cardiac arrest who had admission bilirubin concentrations above 10 μmol/L showed a higher rate of favourable neurological survival at 180 days (OR = 3.02, P = 0.023) (Pudil et al., 2025). This finding is clinically interesting, but it does not demonstrate myocardial protection: admission bilirubin may also reflect hepatic perfusion, haemolysis, or the systemic stress response. Interventional evidence is required before bilirubin can be considered a therapeutic target in this setting.

Antioxidant, inflammatory, vascular, metabolic, and mitochondrial effects may interact in ways that support cardiovascular homeostasis. Reduced ROS and NF-κB-related signalling could limit endothelial injury and inflammatory-cell recruitment while preserving eNOS activity; changes in lipid metabolism could further reduce ox-LDL formation and vascular inflammation (Figure 2). The scheme is therefore a working biological framework rather than a demonstrated causal pathway in humans.

**FIGURE 2:**
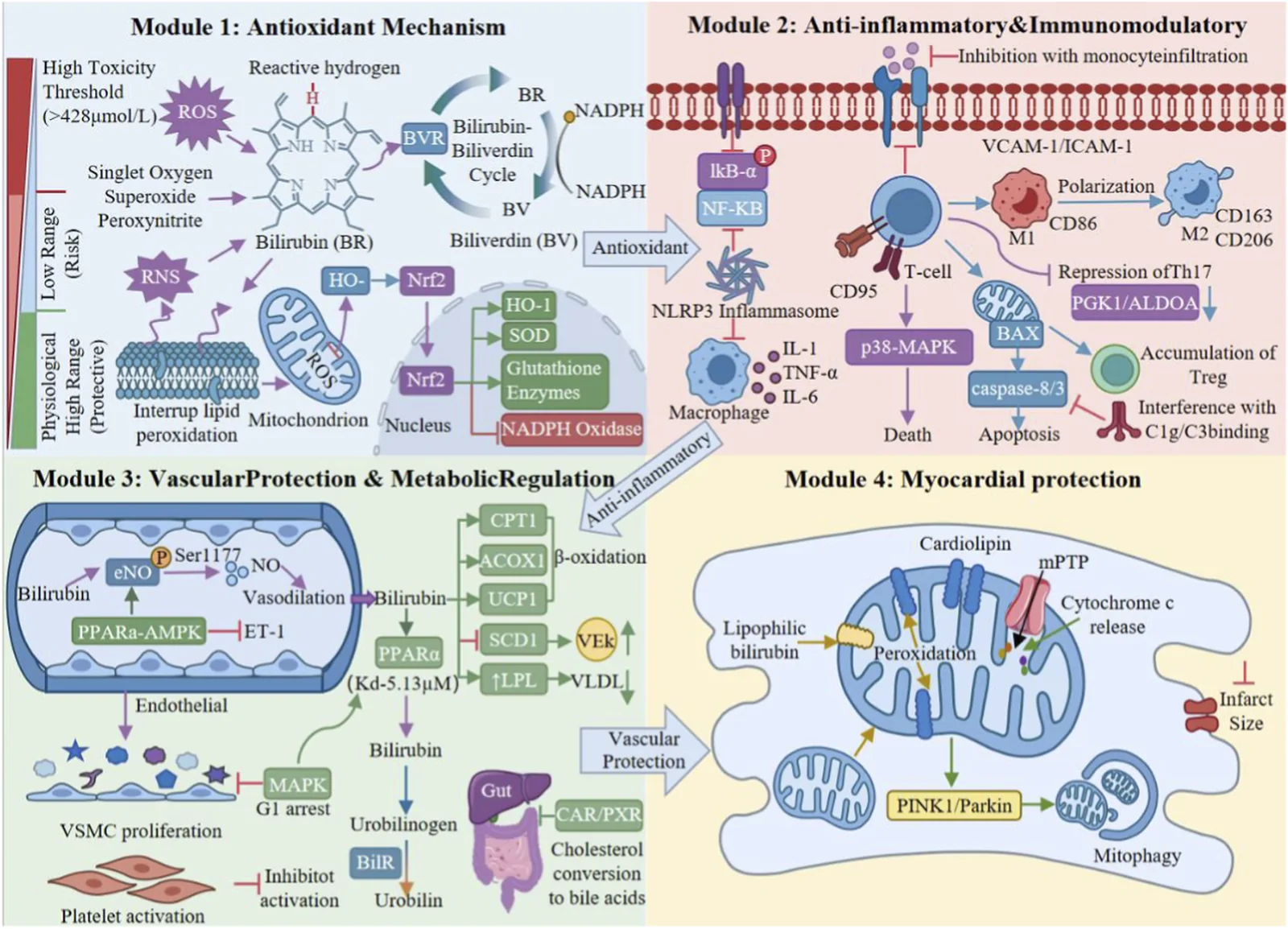
Proposed mechanisms by which bilirubin may protect the cardiovascular system.

When cardiovascular protection is inferred from HO-1 induction rather than direct bilirubin administration or measurement, the contributions of carbon monoxide signalling, iron handling, ferritin induction, and haem removal cannot be separated from those of bilirubin.

This framework provides a rationale for examining whether specific plant metabolites and traditional multi-component formulations modulate HO-1-related cardiovascular responses. Most botanical studies, however, report changes in Nrf2 and/or HO-1 expression without directly measuring bilirubin production. The current literature therefore supports HO-1-associated signalling more strongly than a confirmed bilirubin-mediated mechanism.

## 4 Preclinical cardiovascular evidence for plant metabolites and traditional multi-component formulations relevant to the HO-1/bilirubin axis

The working hypothesis is that selected plant metabolites and traditional multi-component formulations may increase HO-1 expression or activity, enhance haem catabolism, and thereby alter the availability of bilirubin and other haem-derived products. The strength of evidence for this sequence varies markedly. Some studies measured HO activity or circulating bilirubin and used pharmacological or genetic HO-1 interventions; many others reported only Nrf2 and HO-1 expression. The following sections therefore consider both cardiovascular outcomes and the extent to which the proposed HO-1/bilirubin pathway was actually demonstrated.

### 4.1 Curcuminoids and related diarylheptanoids

Curcumin is a non-flavonoid polyphenolic diarylheptanoid and the principal curcuminoid derived from the rhizome of *Curcuma longa* L. [Zingiberaceae]. The major naturally occurring curcuminoids also include demethoxycurcumin (DMC) and bisdemethoxycurcumin (BDMC), which share the diarylheptanoid backbone of curcumin but differ in the number of methoxy groups on their aromatic rings. These structural differences appear to influence their capacity to induce HO-1 and protect vascular cells against oxidative injury.

In human endothelial cells, curcumin, DMC, and BDMC increased HO-1 protein expression and HO enzymatic activity, with curcumin showing the strongest effect, followed by DMC and BDMC. Pretreatment with these curcuminoids also attenuated hydrogen peroxide-induced endothelial-cell injury, whereas the HO inhibitor tin protoporphyrin IX partially reduced their cytoprotective effects. Because HO activity was evaluated by bilirubin formation, this study provides comparative evidence linking curcuminoid structure, HO-1 induction, and endothelial protection. The stronger activity of curcumin suggests that the methoxy-substitution pattern may contribute to the structure–activity relationship of this metabolite class (Jeong et al., 2006).

Curcumin has also shown protective effects in vascular inflammatory models. Xiao et al. reported that curcumin promoted p38 MAPK-dependent Nrf2 activation, increased HO-1 expression and activity, elevated circulating bilirubin, and reduced vascular VCAM-1 and ICAM-1 expression. Pharmacological inhibition of HO activity and local HO-1 silencing attenuated the anti-inflammatory effect, supporting an important functional role for HO-1 in curcumin-mediated vascular protection. These findings provide comparatively strong evidence that activation of the HO-1/haem-catabolic pathway contributes to the vascular anti-inflammatory activity of curcumin (Xiao et al., 2018).

In experimental diabetic cardiomyopathy, curcumin improved mitochondrial function, oxidative-stress indices, myocardial histopathology, and cardiac function. These effects were accompanied by Nrf2 nuclear activation and increased HO-1 expression and activity, while Nrf2 silencing attenuated the cellular protection. This study further supports the involvement of the Nrf2/HO-1 pathway in the cardioprotective actions of curcumin, although downstream haem metabolites were not directly quantified (Wu et al., 2022).

Curcumin is the best-characterised curcuminoid in cardiovascular HO-1 research. Endothelial studies show that DMC and BDMC retain HO-1-inducing activity but are less potent, while vascular and myocardial models link curcumin to antioxidant, anti-inflammatory, and mitochondrial protection. Even so, bilirubin or biliverdin was not measured in every study, and the roles of carbon monoxide, haem removal, and iron handling were not separated. The evidence therefore supports HO-1/haem-catabolic pathway engagement, but not universal bilirubin mediation.

### 4.2 Flavonoids

Flavonoids constitute a structurally diverse class of polyphenolic plant metabolites. Among the compounds discussed in this review, quercetin and isorhamnetin belong to the flavonol subclass, whereas dihydromyricetin is classified as a dihydroflavonol. These metabolites have been investigated in endothelial cells, macrophages, vascular smooth muscle cells, and experimental vascular disease models. Their ability to increase HO-1 expression or stability provides a plausible mechanism through which haem catabolism and downstream biliverdin/bilirubin formation may be enhanced, although these downstream metabolites were not directly measured in most studies.

Quercetin is a widely distributed flavonol and should not be regarded as a metabolite unique to Chinese medicine. In human aortic endothelial cells, quercetin at 5–20 μmol/L dose-dependently reduced lipopolysaccharide-induced oxidant production and the expression of the endothelial adhesion molecules E-selectin and ICAM-1. Quercetin activated p38 MAPK and promoted Nrf2-dependent expression of HO-1, NAD(P)H quinone dehydrogenase 1, and glutamate–cysteine ligase. The p38 inhibitor SB203580 partially attenuated Nrf2 activation and antioxidant-enzyme induction, supporting the participation of the p38/Nrf2/HO-1 pathway in the endothelial response to quercetin (Li et al., 2016).

More direct cardiovascular-cell evidence was subsequently obtained in human coronary artery endothelial cells subjected to hypoxia/reoxygenation injury. Quercetin treatment at 5–20 μmol/L improved cell viability, reduced reactive oxygen species accumulation, preserved mitochondrial function, and decreased mitochondrial apoptosis, accompanied by increased Nrf2 and HO-1 protein expression. Nrf2 silencing attenuated several of these protective effects and reduced downstream HO-1 expression, indicating that Nrf2 activation is functionally important for quercetin-mediated endothelial protection. Together, these studies support an Nrf2-dependent, HO-1-associated vasoprotective effect of quercetin. However, HO-1 itself was not selectively inhibited or genetically silenced, and bilirubin or biliverdin was not quantified; therefore, the contribution of increased bilirubin formation remains a mechanistically plausible but unconfirmed component of quercetin action (Song et al., 2024).

Dihydromyricetin is a dihydroflavonol that has shown particularly strong evidence for direct HO-1 involvement in vascular protection. Earlier experiments in human umbilical-vein endothelial cells demonstrated that dihydromyricetin promoted ERK- and Akt-dependent Nrf2 activation, increased HO-1 expression, and reduced oxidative injury, suggesting that Nrf2/HO-1 signalling contributes to its endothelial cytoprotective effects (Luo et al., 2017). More recently, Ye et al. evaluated dihydromyricetin in vascular smooth muscle cells and in angiotensin II-infused ApoE-deficient mice with abdominal aortic aneurysm. Dihydromyricetin markedly increased HO-1 expression, suppressed vascular smooth muscle-cell inflammation and oxidative stress, preserved the contractile phenotype, and reduced aneurysm formation and elastin degradation. Mechanistically, it promoted HIF-1α-mediated transcription of *HMOX1* rather than relying primarily on Nrf2 and also bound HO-1 at Lys243, thereby limiting ubiquitination-mediated protein degradation and stabilising HO-1.

The functional importance of HO-1 in this model was supported by both pharmacological and genetic interventions. Zinc protoporphyrin IX attenuated the protective effects of dihydromyricetin in vascular smooth muscle cells, whereas vascular smooth muscle cell-specific *Hmox1* knockdown largely abolished its anti-aneurysmal effects *in vivo*. This study therefore provides comparatively strong evidence that HO-1 is required for a substantial proportion of the vascular protection produced by dihydromyricetin. Nevertheless, because bilirubin and biliverdin were not directly quantified, the study demonstrates HO-1-dependent vascular protection rather than definitive bilirubin-mediated protection (Ye et al., 2025).

Isorhamnetin is a methylated flavonol structurally related to quercetin. Its involvement in HO-1-related cardiovascular protection has been investigated in oxidised low-density lipoprotein-treated macrophages and in ApoE-deficient mice with atherosclerosis. In THP-1-derived macrophages, pretreatment with isorhamnetin at 5–20 μmol/L reduced reactive oxygen species generation, lipid accumulation, mitochondrial dysfunction, and apoptosis, while promoting Akt phosphorylation, Nrf2 nuclear translocation, and HO-1 expression. The PI3K inhibitor LY294002 suppressed Akt activation, Nrf2 translocation, and HO-1 induction, whereas zinc protoporphyrin IX attenuated the antioxidant and anti-apoptotic effects of isorhamnetin, supporting functional participation of the PI3K/Akt/Nrf2/HO-1 pathway.

In ApoE-deficient mice fed a high-fat and high-cholesterol diet, oral administration of isorhamnetin at 20 mg/kg/day for 8 weeks reduced aortic atherosclerotic lesion area, macrophage accumulation, caspase-3 expression, and macrophage apoptosis without producing a significant change in circulating lipid concentrations. These results suggest that its anti-atherosclerotic activity was not solely attributable to lipid lowering and was consistent with the antioxidant and anti-apoptotic mechanisms observed *in vitro*. However, because HO-1 inhibition was performed only in the cell experiments, the extent to which HO-1 was required for the reduction in atherosclerotic lesions *in vivo* remains to be established. Direct bilirubin-pathway measurements were also absent (Luo et al., 2015).

Quercetin, dihydromyricetin, and isorhamnetin regulate HO-1-related stress responses through different upstream mechanisms. The dihydromyricetin study provides the strongest direct evidence because it combined pharmacological inhibition with smooth-muscle-cell-specific *Hmox1* knockdown. Quercetin and isorhamnetin provide functional evidence at the level of upstream Nrf2-related pathways, but HO-1 dependence was less completely tested *in vivo*. None of the studies directly quantified bilirubin or biliverdin; participation of these products therefore remains unconfirmed.

### 4.3 Triterpenoid saponins

Triterpenoid saponins constitute an important group of glycosylated plant metabolites with structurally diverse aglycones. The compounds retained in this section include astragaloside IV and the ginsenosides Rg3 and Ro, which have been evaluated in cardiomyocyte, myocardial ischaemia/reperfusion, myocardial hypertrophy, and vascular-repair models. Although these metabolites regulate distinct upstream targets, their reported cardiovascular actions converge partly on Nrf2-dependent induction of HO-1. Increased HO-1 expression or activity may enhance haem catabolism and potentially increase the availability of downstream biliverdin and bilirubin; however, direct measurement of these metabolites remains uncommon in the available saponin studies.

Astragaloside IV is a representative cycloartane-type triterpenoid saponin associated with Astragali Radix. Its involvement in HO-1-related myocardial protection was investigated in H9c2 cardiomyocytes subjected to 12 h of hypoxia followed by 8 h of reoxygenation. Astragaloside IV at 10 and 100 μmol/L increased cell viability and reduced lactate dehydrogenase release, with 100 μmol/L subsequently used for the mechanistic experiments. Treatment reduced reactive oxygen species and malondialdehyde levels, increased superoxide dismutase activity, and decreased the release of interleukin-6 and tumour necrosis factor-α. Pharmacological inhibition of HO activity with zinc protoporphyrin IX partially attenuated these antioxidant and anti-inflammatory effects, whereas cobalt protoporphyrin produced a similar protective response. These results support the functional participation of HO activity in the protection afforded by astragaloside IV against hypoxia/reoxygenation injury (Yang et al., 2019).

Mechanistically, astragaloside IV increased PI3K expression and Akt phosphorylation, promoted nuclear accumulation of Nrf2, reduced nuclear Bach1 expression, and increased HO-1 protein expression. The PI3K inhibitor LY294002 partially reversed these molecular changes, indicating that astragaloside IV may induce HO-1 by altering the balance between the positive transcriptional regulator Nrf2 and the transcriptional repressor Bach1 downstream of PI3K/Akt signalling. This study therefore provides direct cardiovascular-cell evidence for pharmacological HO involvement in astragaloside IV-mediated protection. Nevertheless, the experiments were restricted to an *in vitro* cardiomyocyte model, and neither bilirubin nor biliverdin was quantified (Yang et al., 2019).

Complementary vascular evidence has been obtained in high-glucose-exposed human endothelial progenitor cells and streptozotocin-induced diabetic mice. Astragaloside IV reduced endothelial progenitor-cell apoptosis, improved viability and tube formation, alleviated mitochondrial oxidative damage, and promoted wound repair and CD31 expression in diabetic mice. These effects were accompanied by increased GSK-3β phosphorylation and increased expression of Nrf2, HO-1, and NQO1, whereas pharmacological activation of GSK-3β attenuated the protective response. These findings extend the potential relevance of astragaloside IV from cardiomyocyte stress to vascular repair in diabetes, although HO-1 was not directly inhibited and the experimental endpoint was diabetic wound healing rather than a primary cardiac event (Zou et al., 2025).

Ginsenoside Rg3 is a dammarane-type triterpenoid saponin that has been studied in both myocardial ischaemia/reperfusion and myocardial hypertrophy. In a murine ischaemia/reperfusion model, mice received ginsenoside Rg3 at 5, 10, or 20 mg/kg for 7 days before 30 min of coronary ischaemia and 6 h of reperfusion. The 10- and 20-mg/kg doses improved left ventricular function, reduced infarct size, and alleviated myocardial structural injury. In H9c2 cardiomyocytes subjected to oxygen–glucose deprivation/reoxygenation, ginsenoside Rg3 at 5–20 μmol/L reduced ferrous-iron accumulation, increased glutathione, GPX4, and FTH1, and increased Nrf2 nuclear translocation and the expression of HO-1 and NQO1. The ferroptosis inducer erastin partially attenuated these effects, supporting an anti-ferroptotic component of the myocardial protection (Zhong et al., 2024).

Nrf2 silencing partially abolished the anti-ferroptotic effects of ginsenoside Rg3 and reduced the induction of HO-1 and NQO1, providing functional evidence that Nrf2 is required for a substantial proportion of its cellular protection. Molecular docking further suggested a potential interaction between Rg3 and Keap1, although this computational result should be regarded as supportive rather than definitive evidence of direct binding. In a separate study using angiotensin II-stimulated human cardiomyocytes and rats with transverse-aortic-constriction-induced hypertrophy, Rg3 reduced myocardial hypertrophy, fibrosis, NLRP3 inflammasome activation, and oxidative stress, accompanied by restoration of Nrf2 and HO-1 expression. Inhibition of SIRT1 with AGK2 attenuated the broader protective effects, suggesting that SIRT1-dependent regulation contributes to the anti-hypertrophic activity of Rg3 (Ren et al., 2021; Zhong et al., 2024).

Ginsenoside Ro is an oleanane-type triterpenoid saponin with emerging evidence in myocardial ischaemic injury. In neonatal rat ventricular myocytes subjected to oxygen–glucose deprivation and in murine myocardial-injury models induced by coronary artery ligation or isoproterenol, Ro improved cardiac function, reduced myocardial histopathological injury and apoptosis, and attenuated iron accumulation and lipid peroxidation. Mechanistic experiments identified the branched-chain amino-acid aminotransferases BCAT1 and BCAT2 as upstream molecular targets. Pharmacological inhibition and silencing of BCAT1/2 substantially attenuated the cardioprotective and anti-ferroptotic effects of Ro and reduced activation of the Keap1/Nrf2/HO-1 pathway. These findings provide relatively strong target-level evidence linking Ro to the regulation of ferroptosis and myocardial protection, although HO-1 itself was not independently silenced or inhibited and bilirubin production was not measured (Liu et al., 2025).

The retained saponin studies converge on HO-1-related antioxidant and ferroptosis pathways but differ in mechanistic depth. Astragaloside IV provides pharmacological evidence for HO participation, whereas the Rg3 and Ro studies establish functional roles for upstream Nrf2 or BCAT1/2-related regulation. Because bilirubin and biliverdin were not measured, these effects should be described as HO-1-associated or HO-dependent where directly supported, rather than bilirubin-mediated.

### 4.4 Alkaloids and alkaloid-derived molecules

Alkaloids are a structurally diverse group of nitrogen-containing plant metabolites with reported antioxidant, anti-inflammatory, and cardioprotective activities. The cardiovascular evidence reviewed in this section focuses on berberine and tetramethylpyrazine, whereas DT-010 is discussed separately because it is a synthetic danshensu–tetramethylpyrazine conjugate rather than a naturally occurring alkaloid. Although these molecules act through different upstream regulatory mechanisms, their cardiovascular effects have commonly been associated with increased Nrf2 activity and HO-1 expression.

Berberine is a protoberberine isoquinoline alkaloid that has been investigated in myocardial injury and atherosclerosis models. In experimental doxorubicin-induced cardiotoxicity, berberine reduced oxidative stress, mitochondrial dysfunction, cardiomyocyte apoptosis, and myocardial fibrosis, while increasing nuclear Nrf2 accumulation and the expression of HO-1 and mitochondrial transcription factor A. Pharmacological inhibition of Nrf2 attenuated these protective effects, supporting an Nrf2-dependent antioxidant mechanism in which HO-1 acts as an important downstream component (Wang et al., 2023). In macrophage foam cells and apolipoprotein E-deficient mice, berberine also reduced lipid accumulation and atherosclerotic lesion development through coordinated regulation of scavenger-receptor expression, cholesterol efflux, and Nrf2/HO-1-related signalling (Yang et al., 2020). These findings suggest that berberine may protect the cardiovascular system through the integration of antioxidant, mitochondrial, and lipid-regulatory mechanisms.

Tetramethylpyrazine, also known as ligustrazine, provides comparatively direct evidence for the functional involvement of HO activity in myocardial protection. In a rat myocardial ischaemia/reperfusion model, tetramethylpyrazine increased myocardial HO-1 expression, reduced superoxide generation, and decreased ventricular fibrillation and infarct size. The HO inhibitor zinc protoporphyrin IX substantially weakened its infarct-limiting effect, indicating that HO activity contributes to the cardioprotective actions of tetramethylpyrazine (Chen et al., 2006). These findings support a mechanistic connection between tetramethylpyrazine-induced HO-1 activation, reduced oxidative injury, and improved myocardial tolerance to ischaemia/reperfusion.

DT-010 is a synthetic conjugate of danshensu and tetramethylpyrazine and should therefore be distinguished from tetramethylpyrazine itself. In cardiomyocytes exposed to oxidative injury or hypoxia/reoxygenation, DT-010 reduced intracellular free-radical accumulation and apoptosis while increasing PGC-1α, Nrf2, mitochondrial transcription factor A, and HO-1 expression. In a rat myocardial ischaemia/reperfusion model, DT-010 also reduced myocardial infarct size, supporting a cardioprotective effect associated with activation of the PGC-1α/Nrf2/HO-1 pathway (Zhang et al., 2017). The coordinated regulation of mitochondrial biogenesis and endogenous antioxidant defence may therefore represent an important feature of DT-010-mediated myocardial protection.

Berberine, tetramethylpyrazine, and DT-010 illustrate distinct routes to HO-1-related myocardial or vascular protection. Tetramethylpyrazine provides the clearest evidence for functional HO involvement because HO inhibition weakened its infarct-limiting effect. Evidence for berberine and DT-010 mainly shows upstream Nrf2- or PGC-1α-related signalling accompanied by increased HO-1 expression. Neither bilirubin nor biliverdin was measured, and carbon monoxide, haem removal, and iron handling remain plausible alternative contributors.

### 4.5 Diterpenoid quinones and related derivatives

Diterpenoid quinones constitute a structurally distinct group of lipophilic plant metabolites. Tanshinone IIA is a representative abietane-type diterpenoid quinone naturally occurring in the root and rhizome of *Salvia miltiorrhiza* Bunge [Lamiaceae], whereas sodium tanshinone IIA sulfonate is a water-soluble sulfonated derivative developed to improve its pharmaceutical properties. Current cardiovascular evidence suggests that these molecules can enhance Nrf2/HO-1-related antioxidant responses and protect myocardial or vascular cells under conditions of oxidative stress.

Tanshinone IIA has been investigated in cardiomyocytes subjected to oxygen–glucose deprivation/reoxygenation and in rat models of myocardial ischaemia/reperfusion injury. Treatment reduced cardiomyocyte apoptosis, inflammatory responses, lipid peroxidation, ferrous-iron accumulation, and myocardial injury, accompanied by suppression of HDAC1 and activation of the Nrf2–xCT/GPX4/HO-1 pathway. Computational analyses identified HDAC1 as a potential molecular target of tanshinone IIA, while HDAC1 overexpression attenuated its anti-apoptotic and anti-ferroptotic effects. These findings support a regulatory relationship between HDAC1 suppression, Nrf2 activation, HO-1 expression, and myocardial protection. However, the predicted interaction with HDAC1 should not be described as confirmed direct binding without additional biophysical validation (Yan et al., 2025).

Sodium tanshinone IIA sulfonate has provided complementary evidence in vascular endothelial injury. In human umbilical-vein endothelial cells exposed to homocysteine, sodium tanshinone IIA sulfonate reduced oxidative stress, mitochondrial dysfunction, and cell death while restoring proliferation and migration. These effects were accompanied by activation of AKT/MAPK signalling, increased SIRT1 and Nrf2 activity, and induction of HO-1. Pharmacological inhibition of HO-1 or upstream signalling components partially weakened the endothelial protection, whereas SIRT1 silencing suppressed Nrf2 and HO-1 expression. The study therefore supports functional participation of the SIRT1/Nrf2/HO-1 pathway in the vascular actions of this tanshinone IIA derivative (Zhou et al., 2023).

Tanshinone IIA and sodium tanshinone IIA sulfonate provide complementary myocardial and endothelial evidence for HO-1-related protection. Their studies implicate HDAC1 or SIRT1/Nrf2 regulation, but neither directly quantified bilirubin or biliverdin or used product-specific rescue. The data therefore support HO-1-associated cardiovascular protection while leaving the contribution of bilirubin, carbon monoxide, haem removal, and iron handling unresolved.

The experimental models, intervention regimens, pathway-specific controls, HO-1-related measurements, downstream haem-catabolic products, principal cardiovascular effects, evidence strength, and major limitations of the representative studies discussed in Sections 4.1–4.5 are summarised in Table 1.

**TABLE 1:** Pharmacological characteristics and evidence strength of selected plant metabolites and derivatives in cardiovascular models relevant to the HO-1/bilirubin axis.

Metabolite/derivative	Model and regimen	Mechanistic test	HO-1 axis and cardiovascular effect	Evidence/limitation/reference
4.1 Curcuminoids and related diarylheptanoids				
Curcumin, DMC, BDMC	Human ECs + H_2_O_2_; 10 μmol/L, 6 h	SnPP; CoPP	HO-1 protein/activity ↑; bilirubin-formation assay; cell injury ↓; curcumin > DMC > BDMC	A; *in vitro*; bilirubin-specific mediation not isolated. Jeong et al., 2006
Curcumin	HAECs + rabbit carotid inflammation; 20–100 μmol/L; 0.3% diet, 4 weeks	ZnPP; HO-1 siRNA; Nrf2 siRNA	HO-1/activity ↑; serum bilirubin ↑; VCAM-1/ICAM-1 ↓	A; bilirubin-specific contribution not isolated. Xiao et al., 2018
Curcumin	High-glucose H9c2 + HFD/STZ rats; 100 mg/kg/day, 3 months	Nrf2 shRNA	Nrf2/HO-1/activity ↑; ROS and myocardial injury ↓; cardiac function ↑	C; no HO-1-specific test or bilirubin assay. Wu et al., 2022
4.2 Flavonoids				
Quercetin	HCAEC H/R; 5–20 μmol/L; ∼24 h	Nrf2 siRNA	Nrf2/HO-1 ↑; ROS/apoptosis ↓; viability/mitochondrial function ↑	C; HO-1 and bilirubin not directly tested. Song et al., 2024
Dihydromyricetin	VSMCs + Ang-II/ApoE^−/−^ AAA; 1–20 μmol/L; 125/250 mg/kg/day, 28 d	ZnPPIX; HIF-1α/Nrf2 inhibitors; VSMC-sh*Hmox1*	*HMOX1* transcription and HO-1 stability ↑; inflammation/AAA/elastin loss ↓	B; strong HO-1 causality; bilirubin/biliverdin not measured. Ye et al., 2025
Isorhamnetin	Ox-LDL THP-1 cells + HFC/ApoE^−/−^ mice; 5–20 μmol/L, 8 h; 20 mg/kg/day, 8 weeks	LY294002; ZnPP; lovastatin	PI3K/Akt/Nrf2/HO-1 ↑; ROS/apoptosis/lipid loading and lesion area ↓	B/C; HO-1 inhibition only *in vitro*; bilirubin not measured. Luo et al., 2015
4.3 Triterpenoid saponins				
Astragaloside IV	H9c2 H/R; 0.1–100 μmol/L; key dose 100 μmol/L, 20 h	ZnPP; CoPP; LY294002	PI3K/Akt/Nrf2 ↑; Bach1 ↓; HO-1 ↑; ROS/inflammation/cell injury ↓	B; *in vitro* only; bilirubin/biliverdin not measured. Yang et al., 2019
Ginsenoside Rg3	H9c2 OGD/R + mouse I/R; 5–20 μmol/L, 24 h; 5–20 mg/kg, 7 d	Nrf2 siRNA; erastin	Nrf2/HO-1/NQO1 ↑; Fe2+/ferroptosis/infarct ↓; LVEF/LVFS ↑	C; HO-1 not directly tested; bilirubin not measured. Zhong et al., 2024
Ginsenoside Ro	NRVMs + murine LAD/ISO injury; 5–20 μmol/L; 20–80 mg/kg, 2 weeks	BAY-069; BCAT1/2 siRNA	BCAT1/2 → Keap1/Nrf2/HO-1; Fe2+/ferroptosis ↓; cardiac function ↑	C; HO-1/bilirubin causality not tested. Liu et al., 2025
4.4 Alkaloids and alkaloid-derived molecules				
Berberine	DOX-treated cardiac cells + rats; 1 μmol/L pretreatment; 60 mg/kg, 3 weeks	Nrf2 inhibitor trigonelline	Nrf2/HO-1/TFAM ↑; oxidative/mitochondrial injury, apoptosis and fibrosis ↓	C; no HO-1-specific or bilirubin test. Wang Y. et al., 2023
Tetramethylpyrazine	Rat myocardial I/R; 5/10 mg/kg i.v.; 45-min ischaemia/1-h reperfusion	ZnPP	HO-1 ↑; superoxide, ventricular fibrillation and infarct size ↓	B; no genetic or product-specific confirmation. Chen et al., 2006
DT-010	Cardiomyocyte oxidative/H/R injury + rat I/R; 10–100 μmol/L; 10 mg/kg	No HO-1-specific intervention	PGC-1α/Nrf2/TFAM/HO-1 ↑; ROS/apoptosis/infarct ↓	D; no HO activity or bilirubin measurement. Zhang et al., 2017
4.5 Diterpenoid quinones and related derivatives				
Tanshinone IIA	Myocardial cells OGD/R + rat I/R; 10 μmol/L; 20 mg/kg i.v	HDAC1 overexpression	HDAC1 ↓ → Nrf2/xCT/GPX4/HO-1 ↑; Fe2+/ferroptosis/infarct ↓	C; HO-1 causality not directly tested. Yan et al., 2025
Sodium tanshinone IIA sulfonate	HUVECs + homocysteine; dose and duration not reported in the accessible source	Pathway inhibitors; SIRT1/NNMT siRNA	SIRT1/Nrf2/HO-1 ↑; ROS/mitochondrial injury ↓; survival/migration ↑	B/C; cell model; regimen incompletely reported. Zhou et al., 2023

HO-1, induction alone was not interpreted as proof of increased bilirubin production or bilirubin-mediated cardiovascular protection. Evidence categories: A, direct HO-1/bilirubin-pathway evidence, based on HO-1-specific intervention with direct bilirubin measurement or HO, activity assessed through bilirubin formation; B, direct HO/HO-1, dependence without bilirubin measurement; C, functional dependence on an upstream regulator with HO-1, as a downstream readout; and D, associative HO-1, evidence without pathway-specific intervention. Abbreviations: AAA, abdominal aortic aneurysm; Ang-II, angiotensin II; BDMC, bisdemethoxycurcumin; CoPP, cobalt protoporphyrin; DMC, demethoxycurcumin; DOX, doxorubicin; ECs, endothelial cells; H/R, hypoxia/reoxygenation; HAECs, human aortic endothelial cells; HCAECs, human coronary artery endothelial cells; HUVECs, human umbilical-vein endothelial cells; I/R, ischaemia/reperfusion; ISO, isoproterenol; LAD, left anterior descending coronary artery; LVEF, left-ventricular ejection fraction; LVFS, left-ventricular fractional shortening; NRVMs, neonatal rat ventricular myocytes; OGD/R, oxygen-glucose deprivation/reoxygenation; SnPP/ZnPP/ZnPPIX, metalloporphyrins used to inhibit or modulate HO, activity; TFAM, mitochondrial transcription factor A; VSMCs, vascular smooth-muscle cells.

### 4.6 Traditional multi-component formulations used in Chinese medicine

Multi-component formulations differ from isolated metabolites because several constituents may act on overlapping or complementary targets. The five retained studies used models of myocardial ischaemia/reperfusion, hypoxia/reoxygenation, post-infarction hypertrophy, and disturbed iron homeostasis. All reports provided complete formula compositions, but authentication, batch documentation, preparation, and quality control were reported to different standards. Supplementary Table S2 maps the reported material names to recognised source taxa or source classes, families, and medicinal parts. Where an article did not identify the exact species or provide voucher-based authentication, the mapping is a nomenclatural aid rather than retrospective confirmation of the experimental material. Table 2 compares the study designs, principal HO-1-related findings, and evidence limitations.

**TABLE 2:** Cardiovascular evidence for traditional multi-component formulations relevant to the HO-1/bilirubin axis.

Formulation	Model and regimen	Main HO-1-related findings	Evidence interpretation/limitation/reference
Dingxin Recipe I	Wistar rat MIRI; 9.375, 18.75, or 37.5 g/kg/day orally for 7 d before 30-min LAD occlusion and 1-h reperfusion; amiodarone positive control	Nrf2, HO-1, Bcl-2, GSH, and SOD ↑; p-GSK3β, Bax, CK-MB, LDH, cTnI, MDA, and histological injury ↓	D: associative GSK3β/Nrf2/HO-1 evidence. No pathway-specific intervention, HO activity, bilirubin, or biliverdin measurement. Liu et al., 2020
Wenyang Tongmai formula	H9c2 CoCl_2_-based H/R; medicated serum from rats given 13 g/kg every 12 h for 3 d; 5%, 10%, or 20% serum; 4-h pretreatment, 22-h chemical hypoxia, and 2-h reoxygenation	Cell viability, SOD, Nrf2, and HO-1 ↑; MDA, LDH, ROS, apoptosis, and HIF-1α ↓	D: associative medicated-serum cell evidence. Active constituents and cellular exposure undefined; no pathway-specific intervention or bilirubin measurement. Ma and Shi, 2022
Wutou Chishizhi Pill	Rat post-MI hypertrophy; 28 d remodelling after LAD ligation, followed by 4.9 g/kg/day orally for 28 d; perindopril positive control	Nrf2, HO-1, NQO1, Bcl-2, and SOD ↑; Keap1, Bax, hypertrophy, fibrosis, ROS, MDA, and apoptosis ↓	D: associative Keap1/Nrf2/HO-1/NQO1 evidence. No pathway-specific intervention or bilirubin measurement; *Aconitum* processing and safety require careful control. Wang X. et al., 2023
Yixin formula	Wistar rat MIRI; 10 mL/kg/day orally (13.69 g/kg/day) for 7 d after ischaemia/reperfusion; EX527 and SRT1720 used with the formula	LVEF, LVFS, SOD, SIRT1, Nrf2, HO-1, and Bcl-2 ↑; myocardial injury, ROS, MDA, H_2_O_2_, SDH, and apoptosis ↓; EX527 weakened and SRT1720 enhanced several effects	C: functional evidence for upstream SIRT1 involvement, but not direct HO-1 dependence. No HO activity, bilirubin, or biliverdin measurement. Dong et al., 2024
Conggan Zhixin formula	Male SD rat MIRI; 30-min LAD occlusion and 120-min reperfusion; 5.32 g/kg/day orally for 14 d after surgery; Shexiang Baoxin Pill and diltiazem comparators	Nrf2, HO-1, and Heph mRNA/protein ↑; DMT1 and myocardial iron deposition ↓; myocardial and mitochondrial morphology improved	D: associative HO-1/iron-transport evidence. No pathway-specific intervention or bilirubin measurement; contributions of HO-1 and altered iron transport were not separated. Wang X. et al., 2025

Composition, preparation, and quality-control information are reported in Section 4.6 and are not repeated in the table. Evidence categories follow Table 1; the formulation studies included here were classified as C or D.

Dingxin Recipe I contained Salviae Miltiorrhizae Radix et Rhizoma, Notoginseng Radix et Rhizoma, Sophorae Flavescentis Radix, Coptidis Rhizoma, Ziziphi Spinosae Semen, Codonopsis Radix, Ganoderma, Poria, Trichosanthis Fructus, and Paeoniae Radix Rubra. The authenticated materials were prepared as a water decoction containing 3.75 g crude drug/mL, although individual ratios and chemical quality-control markers were not reported. Wistar rats received 9.375, 18.75, or 37.5 g/kg/day for 7 days before 30 min of coronary occlusion and 1 h of reperfusion. The middle and high doses reduced myocardial injury and oxidative-stress markers, increased Nrf2, HO-1, and Bcl-2, and reduced p-GSK3β and Bax. Because GSK3β and HO-1 were not directly manipulated, the findings show an association with GSK3β/Nrf2/HO-1 signalling rather than pathway dependence or bilirubin mediation (Liu et al., 2020).

Wenyang Tongmai Formula contained Aurantii Fructus Immaturus 12 g, Allii Macrostemonis Bulbus 9 g, Cinnamomi Ramulus 3 g, Magnoliae Officinalis Cortex 12 g, Trichosanthis Fructus 15 g, Ginseng Radix et Rhizoma 12 g, and Notoginseng Radix et Rhizoma 9 g. Batch numbers, supplier information, and stated quality-control compliance were reported for the formula granules. Medicated serum was obtained from rats given 13 g/kg every 12 h for 3 days and tested at 5%, 10%, or 20% in CoCl_2_-treated H9c2 cells. The serum improved viability, redox indices, and apoptosis and altered HIF-1α, Nrf2, and HO-1 expression. Because chemical hypoxia and medicated serum were used, the active circulating constituents and their cellular exposure remain undefined (Ma and Shi, 2022).

Wutou Chishizhi Pill contained Aconiti Radix 5 g, Aconiti Lateralis Radix Praeparata 5 g, Zanthoxyli Pericarpium 10 g, Zingiberis Rhizoma 5 g, and the mineral drug Halloysitum Rubrum 10 g; the water decoction contained 0.72 g crude drug/mL. After 28 days of post-infarction remodelling, rats received 4.9 g/kg/day for a further 28 days. Treatment reduced hypertrophy, fibrosis, oxidative stress, and apoptosis, with lower Keap1 and higher Nrf2, HO-1, NQO1, and Bcl-2 expression. No pathway-specific intervention was used. The presence of *Aconitum*-derived drugs also makes processing control and toxicological evaluation particularly important (Wang X. et al., 2023).

Yixin Formula contained Astragali Radix 15 g, Chuanxiong Rhizoma 12 g, Salviae Miltiorrhizae Radix et Rhizoma 12 g, Rhodiolae Crenulatae Radix et Rhizoma 10 g, Angelicae Sinensis Radix 9 g, Cyperi Rhizoma 9 g, and Cinnamomi Ramulus 6 g. Geographical origins and batch numbers were reported, the granules were stated to comply with the 2020 Chinese Pharmacopoeia, and the final preparation contained 1.369 g crude drug/mL. Rats received 10 mL/kg/day for 7 days after myocardial ischaemia/reperfusion. EX527 weakened several functional, oxidative, and apoptotic effects, whereas SRT1720 enhanced them, supporting a role for SIRT1 upstream of Nrf2 and HO-1. The study did not directly establish HO-1 dependence (Dong et al., 2024).

Conggan Zhixin Formula contained Ginseng Radix et Rhizoma Rubra 10 g, Angelicae Sinensis Radix 10 g, Salviae Miltiorrhizae Radix et Rhizoma 10 g, Bupleuri Radix 10 g, Curcumae Longae Rhizoma 10 g, Curcumae Radix 10 g, Sinapis Semen 5 g, and the animal-derived drug *Aspongopus* 5 g. The water decoction contained 1.5 g crude drug/mL. After 30 min of coronary occlusion and 120 min of reperfusion, rats received 5.32 g/kg/day for 14 days. Treatment improved myocardial and mitochondrial morphology, reduced iron deposition, increased Nrf2, HO-1, and hephaestin, and decreased DMT1. The study links HO-1-associated signalling with iron transport but cannot separate their respective contributions (Wang X. et al., 2025).

Across the five studies, changes in HO-1-related proteins accompanied improvements in oxidative injury, apoptosis, remodelling, or iron dysregulation. Yixin Formula had the strongest mechanistic design because an upstream regulator was pharmacologically modulated; the other studies relied on parallel changes in pathway proteins and outcomes. None measured HO activity, bilirubin, or biliverdin, directly manipulated HO-1, or used product-specific rescue. These data support formulation-associated HO-1 responses, not a confirmed HO-1/bilirubin-mediated mechanism.

## 5 Discussion: integrating bilirubin biology with botanical cardiovascular evidence

### 5.1 Biological coherence between bilirubin actions and the reviewed interventions

The bilirubin literature describes a coherent set of antioxidant, inflammatory, endothelial, metabolic, and mitochondrial actions that could contribute to cardiovascular protection. Many outcomes reported for the plant metabolites and formulations reviewed here—lower oxidative stress and inflammation, improved endothelial or myocardial function, reduced apoptosis and remodelling, and modulation of ferroptosis—fit within this biological framework (Maruhashi et al., 2019; Ai et al., 2021; Kim et al., 2024). This coherence is useful for hypothesis generation, but it is not evidence that bilirubin mediated each intervention’s effects.

Most evidence for bilirubin itself comes from biochemical, cellular, and animal studies, while human data are mainly observational. HO-1 induction also has consequences beyond bilirubin: it removes pro-oxidant haem and generates biliverdin, carbon monoxide, and ferrous iron in parallel. Cardiovascular effects may therefore reflect different combinations of bilirubin/biliverdin redox activity, carbon monoxide signalling, haem removal, and adaptive iron handling (Ryter et al., 2006; Abraham and Kappas, 2008; Gáll et al., 2019).

### 5.2 Strength and limitations of the current botanical evidence

HO-1 recurred across the cardiovascular studies, but causal evidence was uneven. Curcumin provided the most complete evidence of HO-1/haem-catabolic pathway engagement because HO activity, circulating bilirubin, pharmacological inhibition, and local HO-1 silencing were assessed in a vascular-inflammation model (Xiao et al., 2018). Dihydromyricetin showed HO-1-dependent vascular protection through both pharmacological inhibition and smooth-muscle-cell-specific *Hmox1* knockdown, although bilirubin was not measured (Ye et al., 2025). Tetramethylpyrazine and astragaloside IV provide pharmacological evidence for HO involvement. Most other metabolites and all five formulations show upstream pathway changes together with increased HO-1 expression, but no direct test of HO-1 dependence.

The unresolved step is the transition from HO-1 induction to bilirubin-mediated protection. *HMOX1* transcription and HO-1 protein abundance do not necessarily indicate greater enzymatic flux or bilirubin availability. A rise in circulating bilirubin may also result from altered hepatic uptake, UGT1A1-mediated conjugation, biliary excretion, haemolysis, or intestinal metabolism (Ma et al., 2017; Vítek and Tiribelli, 2021). Most studies did not quantify bilirubin or biliverdin, manipulate *BVRA*, or use bilirubin rescue. The current evidence is therefore stronger for intervention-induced HO-1-related responses than for the complete sequence of HO-1 activation, bilirubin formation, and bilirubin-dependent cardiovascular protection.

Design limitations add further uncertainty. Much of the evidence comes from cultured cells or short-term rodent models, often with pretreatment rather than treatment after disease establishment. Some *in vitro* concentrations may exceed clinically achievable unbound exposure, and reporting of randomisation, blinding, sample-size justification, exclusions, and attrition was inconsistent. Differences in models, dosing, assays, and outcome definitions also preclude direct comparisons. The categories in Tables 1 and 2 are therefore qualitative mechanistic classifications, not formal risk-of-bias grades.

### 5.3 Reproducibility, pharmacokinetics, and safety

Translation depends on precise definition of the investigated material. Isolated metabolites should be reported with identity, purity, stereochemistry where relevant, source, vehicle, and stability. Botanical drugs and traditional multi-component formulations require accepted scientific names and medicinal parts for botanical constituents; recognised pharmacopoeial names for mineral or animal-derived materials; processing methods; complete composition and ratios; extraction procedures and yield; batch information; voucher specimens or equivalent authentication; chemical fingerprints; quantitative markers; and contaminant testing. All five formulation studies reported complete compositions, but the detail provided for authentication, batches, preparation, and chemical quality control varied, limiting cross-study reproducibility and formulation-specific interpretation (Heinrich et al., 2022). These gaps are summarised in Supplementary Table S2.

Pharmacokinetics is equally important because the parent compound used in cell experiments may not be the principal circulating or tissue-exposed species after oral dosing. Experimentally active concentrations should be compared with measured unbound plasma and cardiovascular tissue exposure and with evidence of HO-1-network engagement. For traditional multi-component formulations, metabolomics, fractionation–reconstitution, and exposure–response analyses may help distinguish absorbed active constituents from quality-control markers (Atanasov et al., 2021). Safety assessment should include bilirubin fractions and hepatic, haemolytic, renal, and haematological indices, because excessive unconjugated bilirubin is toxic and changes in bilirubin may reflect disease rather than beneficial pathway activation (Adin, 2021; Nocentini et al., 2022). Herb–drug interactions require particular attention in patients receiving antiplatelet, anticoagulant, lipid-lowering, antihypertensive, or antidiabetic therapy. For Wutou Chishizhi Pill, rigorous processing control, chemical standardisation, dose justification, and toxicological evaluation are essential because the formulation contains *Aconitum*-derived drugs.

### 5.4 Priorities for mechanistic validation and clinical translation

Mechanistic studies should begin with standardised materials and exposure-confirmed dosing. *HMOX1* inhibition or tissue-specific deletion should then be combined with *BVRA* manipulation, direct HO activity assays, and quantitative measurement of bilirubin and biliverdin. Stable-isotope-labelled haem tracing and LC–MS/MS could distinguish increased production from reduced clearance. Bilirubin or biliverdin rescue would test product-specific causality, while validated carbon monoxide probes and measurements of labile iron, ferritin, ferroportin, hepcidin, lipid peroxidation, and ferroptosis rescue would clarify other branches of haem catabolism. Cardiovascular relevance should be confirmed in models that include important comorbidities, both sexes where appropriate, treatment after disease onset, and reporting consistent with ARRIVE 2.0 (Percie du Sert et al., 2020).

Clinical development should be staged. Early studies should establish product identity and composition, systemic exposure, tolerability, bilirubin fractions, hepatic and haemolysis indices, and potential drug interactions. Proof-of-mechanism studies could then assess endothelial function, oxidative and inflammatory markers, HO-1-related biomarkers, and selected carbon monoxide or iron-homeostasis endpoints. Trials of clinical cardiovascular outcomes would be justified only after material consistency, pharmacokinetics, safety, and pathway engagement have been demonstrated.

Once regarded mainly as a waste product of haem catabolism, bilirubin has emerged as a biologically active molecule with plausible antioxidant, anti-inflammatory, vascular, metabolic, and myocardial effects. The HO-1/bilirubin axis therefore provides a relevant framework for examining how selected plant metabolites and traditional multi-component formulations used in Chinese medicine may influence cardiovascular stress responses. Only a small proportion of studies, however, directly connect HO-1 activation, increased bilirubin production, and cardiovascular protection. Current evidence supports HO-1-associated—and, in fewer cases, HO-1-dependent—protection more strongly than bilirubin-mediated protection. Future progress will depend on standardised materials, exposure-confirmed dosing, direct measurement of haem-catabolic products, pathway-specific manipulation, and product-rescue experiments.
