# Supplementary material for: The HO-1/bilirubin axis in cardiovascular protection: critical evidence for modulation by plant metabolites and traditional multi-component formulations used in Chinese medicine
Source: Front Pharmacol. 2026 Sep 21;17:1902935. doi: 10.3389/fphar.2026.1902935 (PMC13636427; doi:10.3389/fphar.2026.1902935)
Supplement: Supplementary file 1 [file Table2.docx]

**Supplementary Table S2. Nomenclatural mapping, medicinal parts or source classes, and reporting status of ingredients in the five traditional multi-component formulations**

This table maps the pharmacopoeial or material names reported in the original studies to their recognised source taxon(s) or source classes. Where a pharmacopoeial drug may be derived from more than one source taxon and the original study did not identify the exact species, all relevant source taxa are listed and the study-level status is recorded as not reported. The mapping does not substitute for voucher specimens or authentication records from the original experiments.

| **Reported material name** | **Source taxon(s) / source class** | **Family / source class** | **Medicinal part or material** | **Formulation(s)** | **Reporting status and limitation** |
| --- | --- | --- | --- | --- | --- |
| Salviae Miltiorrhizae Radix et Rhizoma | Salvia miltiorrhiza Bunge | Lamiaceae | Root and rhizome | Dingxin Recipe I; Yixin Formula; Conggan Zhixin Formula | Ingredient listed; species-level voucher/authentication details incomplete or not reported. |
| Notoginseng Radix et Rhizoma | Panax notoginseng (Burkill) F.H.Chen | Araliaceae | Root and rhizome | Dingxin Recipe I; Wenyang Tongmai Formula | Ingredient listed; species-level source documentation/voucher not reported. |
| Sophorae Flavescentis Radix | Sophora flavescens Aiton | Fabaceae | Root | Dingxin Recipe I | Ingredient listed; voucher and chemical markers not reported. |
| Coptidis Rhizoma | Coptis chinensis Franch.; Coptis deltoidea C.Y.Cheng & P.K.Hsiao; Coptis teeta Wall. | Ranunculaceae | Rhizome | Dingxin Recipe I | Exact source species not reported; multi-source pharmacopoeial drug. |
| Ziziphi Spinosae Semen | Ziziphus jujuba Mill. var. spinosa (Bunge) Hu ex H.F.Chow | Rhamnaceae | Mature seed | Dingxin Recipe I | Ingredient listed; voucher/source record not reported. |
| Codonopsis Radix | Codonopsis pilosula (Franch.) Nannf.; Codonopsis tangshen Oliv.; Codonopsis pilosula var. modesta (Nannf.) L.T.Shen | Campanulaceae | Root | Dingxin Recipe I | Exact source species not reported; multi-source pharmacopoeial drug. |
| Ganoderma | Ganoderma lingzhi Sheng H.Wu, Y.Cao & Y.C.Dai and/or Ganoderma sinense J.D.Zhao, L.W.Hsu & X.Q.Zhang | Ganodermataceae (fungus) | Fruiting body | Dingxin Recipe I | Reported only as Ganoderma; exact fungal species and voucher not reported. |
| Poria | Wolfiporia cocos (F.A.Wolf) Ryvarden & Gilb. | Polyporaceae (fungus) | Sclerotium | Dingxin Recipe I | Ingredient listed; voucher/source record not reported. |
| Trichosanthis Fructus | Trichosanthes kirilowii Maxim.; Trichosanthes rosthornii Harms | Cucurbitaceae | Mature fruit | Dingxin Recipe I; Wenyang Tongmai Formula | Exact source species not reported; multi-source pharmacopoeial drug. |
| Paeoniae Radix Rubra | Paeonia lactiflora Pall.; Paeonia veitchii Lynch | Paeoniaceae | Root | Dingxin Recipe I | Exact source species not reported; multi-source pharmacopoeial drug. |
| Aurantii Fructus Immaturus | Citrus aurantium L. and permitted cultivars | Rutaceae | Immature fruit | Wenyang Tongmai Formula | Drug name and dose reported; exact cultivar/species-level source and voucher not reported. |
| Allii Macrostemonis Bulbus | Allium macrostemon Bunge; Allium chinense G.Don | Amaryllidaceae | Bulb | Wenyang Tongmai Formula | Exact source species not reported; multi-source pharmacopoeial drug. |
| Cinnamomi Ramulus | Cinnamomum cassia (L.) J.Presl | Lauraceae | Young twig | Wenyang Tongmai Formula; Yixin Formula | Ingredient listed; batch information reported for granules in some studies, but voucher/source identity incomplete. |
| Magnoliae Officinalis Cortex | Magnolia officinalis Rehder & E.H.Wilson; Magnolia officinalis var. biloba Rehder & E.H.Wilson | Magnoliaceae | Stem, root, or branch bark | Wenyang Tongmai Formula | Exact source taxon not reported; multi-source pharmacopoeial drug. |
| Ginseng Radix et Rhizoma | Panax ginseng C.A.Mey. | Araliaceae | Root and rhizome | Wenyang Tongmai Formula | Drug name and dose reported; voucher/source documentation not reported. |
| Aconiti Radix | Aconitum carmichaelii Debeaux | Ranunculaceae | Mother root | Wutou Chishizhi Pill | Drug and dose reported; source authentication and alkaloid standardisation not fully reported. |
| Aconiti Lateralis Radix Praeparata | Aconitum carmichaelii Debeaux | Ranunculaceae | Processed lateral root | Wutou Chishizhi Pill | Processing is critical; detailed processing and toxic-alkaloid control require explicit reporting. |
| Zanthoxyli Pericarpium | Zanthoxylum bungeanum Maxim.; Zanthoxylum schinifolium Siebold & Zucc. | Rutaceae | Mature pericarp | Wutou Chishizhi Pill | Exact source species not reported; multi-source pharmacopoeial drug. |
| Zingiberis Rhizoma | Zingiber officinale Roscoe | Zingiberaceae | Dried rhizome | Wutou Chishizhi Pill | Drug and dose reported; voucher/source documentation not reported. |
| Halloysitum Rubrum | Halloysite-rich hydrated aluminium silicate mineral material | Mineral drug | Mineral material | Wutou Chishizhi Pill | Material named and dosed; mineralogical identity, elemental profile, and contaminant testing incompletely reported. |
| Astragali Radix | Astragalus mongholicus Bunge; Astragalus membranaceus (Fisch.) Bunge | Fabaceae | Root | Yixin Formula | Pharmacopoeial compliance and batch/origin reported; exact source taxon/voucher not stated. |
| Chuanxiong Rhizoma | Ligusticum chuanxiong Hort. | Apiaceae | Rhizome | Yixin Formula | Pharmacopoeial drug name, origin, and batch reported; voucher and taxonomic authentication not stated. |
| Rhodiolae Crenulatae Radix et Rhizoma | Rhodiola crenulata (Hook.f. & Thomson) H.Ohba | Crassulaceae | Root and rhizome | Yixin Formula | Origin and batch reported; voucher/authentication procedure not stated. |
| Angelicae Sinensis Radix | Angelica sinensis (Oliv.) Diels | Apiaceae | Root | Yixin Formula; Conggan Zhixin Formula | Ingredient listed; detailed voucher/authentication and quantitative markers not reported. |
| Cyperi Rhizoma | Cyperus rotundus L. | Cyperaceae | Rhizome | Yixin Formula | Origin and batch reported; voucher/authentication procedure not stated. |
| Ginseng Radix et Rhizoma Rubra | Panax ginseng C.A.Mey. | Araliaceae | Steamed and dried root and rhizome | Conggan Zhixin Formula | Ingredient and dose reported; processing specifications and ginsenoside markers not reported. |
| Bupleuri Radix | Bupleurum chinense DC.; Bupleurum scorzonerifolium Willd. | Apiaceae | Root | Conggan Zhixin Formula | Exact source species not reported; multi-source pharmacopoeial drug. |
| Curcumae Longae Rhizoma | Curcuma longa L. | Zingiberaceae | Rhizome | Conggan Zhixin Formula | Ingredient and dose reported; voucher and quantitative markers not reported. |
| Curcumae Radix | Curcuma wenyujin Y.H.Chen & C.Ling; Curcuma kwangsiensis S.G.Lee & C.F.Liang; Curcuma phaeocaulis Valeton; Curcuma longa L. | Zingiberaceae | Tuberous root | Conggan Zhixin Formula | Exact source species not reported; multi-source pharmacopoeial drug. |
| Sinapis Semen | Sinapis alba L. | Brassicaceae | Mature seed | Conggan Zhixin Formula | Ingredient and dose reported; voucher/source documentation not reported. |
| Aspongopus (Jiuxiangchong) | Coridius chinensis (Dallas, 1851) (syn. Aspongopus chinensis Dallas, 1851) | Dinidoridae (animal-derived) | Dried whole insect | Conggan Zhixin Formula | Animal-derived material named and dosed; specimen identity, origin, batch, and contaminant testing not reported. |

Abbreviations: NR, not reported. Source-taxon mapping should be interpreted as a nomenclatural aid for evaluating reporting quality; it does not establish which source taxon was actually used when the original report did not provide species-level authentication. Pharmacopoeial material names, source taxa, taxonomic authorities, families, and medicinal parts were checked against the authoritative pharmacopoeial and taxonomic sources cited in the Supplementary References.
